# Mr. Freake on the Use of Humulus in Gout

**Published:** 1813-04

**Authors:** 


*For the Medical and Physical Journal.*

MR. FREAKE has just recovered from a violent paroxysm of acute inflammatory Gout, during which he has proved on himself the important fact, that, by taking the Humulus or Hop, immediately on the accession of the fit, and increasing the number of doses so as to exceed the extreme quantity previously recommended, the most salutary effects have been produced, as the annexed Case will explain, which Mr. F. has printed, as an additional proof of the safety of the medicine, and for the information of medical gentlemen, particularly those who, though they have often been told the contrary, still think the inflammatory state of Gout is not that in which Humulus should be administered. Mr. Freake's plan has been to give this medicine early, to assist in expelling Gout from the system, and to prevent the patient from becoming weakened by the disease. The Case is also printed with a hope that suffering individuals will now be relieved, and not be allowed to linger on with disease when there is a *safe* remedy offered them in the Humulus. Mr. F. has used every laudable and honorable means to introduce this medicine into general use for arthritic complaints, during the last eight years, having watched its effects for the four years preceding, and is sorry that he is obliged to admit, that a variety of obstructing causes have hitherto occurred to prevent the completion of his object, but trusts they will now be done away, and this valuable medicine acquire its merited importance.

It is a curious fact, that the patients who had taken the Humulus with beneficial effect previous to taking the Eau Medicinale, have found it necessary, after taking that medicine, to persist in the use of the Humulus for double the time, in order to produce its usual good effects.

It should also be noticed, that it is also in Chronic Cases of Gout that it is necessary to continue the Humulus as an alterative for several months, for in acute Gout the time necessary to relieve is comparatively very short, as the present Case fully proves.

Physicians and others have hinted, that gouty subjects, supposed to have deep-seated chronic affections, would be very happy if they could bring on a fit of the Gout; for the state of debility, and want of action in the system, induced by that disease, is such, that life becomes a burthen to them. Mr.F. does not hesitate to declare, that, if the Humulus were properly administered to such patients (provided no vital organ was otherwise diseased), that either a regular fit of Gout

Gout would be produced, or the patient would be restored to health again.

*Case.*—On Monday night, the 8th of February, 1813, Mr. F. retired to rest between eleven and twelve o'clock. At one he awoke with an acute darting pain in the joint of his left great toe, and at the heel of his right leg, which continued so as to prevent his resting until four o'clock, when he fell asleep, and got into a free perspiration. At five he was disturbed by the father of a young lady who was dangerously ill, and he was obliged to rise in that state, and walk more than half a mile; this increased Mr. F.'s sufferings considerably, and it was with difficulty he got to the house, and afterwards went through the business of the day; in the evening, the lameness increasing, with fever, thirst, dry tongue, and pulse 108; he took a dose of calomel and a saline draught, with a full dose of the tincture and extract of Humulus, and on the Wednesday morning an aperient draught, with a similar dose of the Humulus. These together operated freely without griping or inconvenience; at noon the dose of the Humulus was repeated with a saline draught, and again in the evening, and at night, after which the pain in the joint of the left great toe subsided, but that of the right leg increased to a violent degree, with inflammation, some swelling, and a shining surface, which rendered the limb useless, for he could not bear it to touch the floor. The violence of the pain caused Mr. F. to take more than the *greatest* quantity of the Humulus he had ever given to a patient, by one scruple of the extract and two drachms of the tincture in the twenty-four hours, thus continuing to take four scruples of the extract and one ounce of the tincture daily, until Saturday the 13th, when he got ease, and was able to put his foot to the ground. The fever having subsided, the pulse was reduced to 76, and the tongue was perfectly clear; he then lessened the quantity of the Humulus one-fourth in each day. On Sunday he took another aperient draught with the Humulus, and walked up and down stairs five or six times, which rather fatigued him, and prevented his going out on Monday, as he hoped to do. On Monday night the limb swelled considerably, but he rested remarkably well, and the swelling subsided. He arose on Tuesday so much better, that he resumed his visits to his patients, being (with the exception of weakness from the tenderness of the part) in perfect health, and on Friday, being only the tenth day from the attack, he was able to wear his ordinary shoe without inconvenience.

---

In

In 1805, Mr. F. published an account of the virtues of the Hop, in the Medical and Physical Journal for May; wherein, at page 435, is the following remark: "And, as it has been hinted by a Medical Gentleman of some notoriety, that the Hop is not a good medicine in the Gout, but on the contrary is one of the causes that produce it, I can only say, that, should the legislature be inclined to offer a reward for the discovery of the best medicine to ease, relieve, and prevent the violent recurrence of that complaint, I should not be fearful of gaining the prize by naming the Humulus."

In 1806, Mr. F. had a pamphlet printed, which he presented to most of the Physicians and Surgeons in London, with observations, experiments, and cases; but did not allow any of this edition to be sold.

In 1807, Mr. F. published a second edition, with observations, cases, &c. which was sold by Mr. Highley, and other booksellers.

In 1810, a physician that had seen one of Mr. F.'s patients in March, who had recovered the use of his limbs after being long in a crippled state, and had also just recovered from a paroxysm of acute Gout, and was able to walk out in nine days from the attack, by taking the Humulus, stated in his publication the following May, that "the afflicted have ever had to lament, that medical science had contributed no effectual means to alleviate their sufferings;" this statement induced Mr. F. to publish six additional successful cases, of which that patient's formed one. This pamphlet was likewise sold by Mr. Highley, and other booksellers.

